# Supplementary material for: Spatial Variation in Stream-Water Bacterial Communities Across Sites Differing in Wild Chinese Giant Salamander Detection Frequency
Source: Biology (Basel). 2026 Sep 18;15(18):1647. doi: 10.3390/biology15181647 (PMC13604818; doi:10.3390/biology15181647)
Supplement: Supplementary file 1 [file biology-15-01647-s001.zip › Data Related to “Spatial Variation in Stream-Water Bacterial Communities across Sites Differing in Wild Chinese Giant Salamander Detection Frequency”/Microbial Data/result/03.TaxonVisual/ternary/sample/species/S1-S2-S4.ternary.pdf]

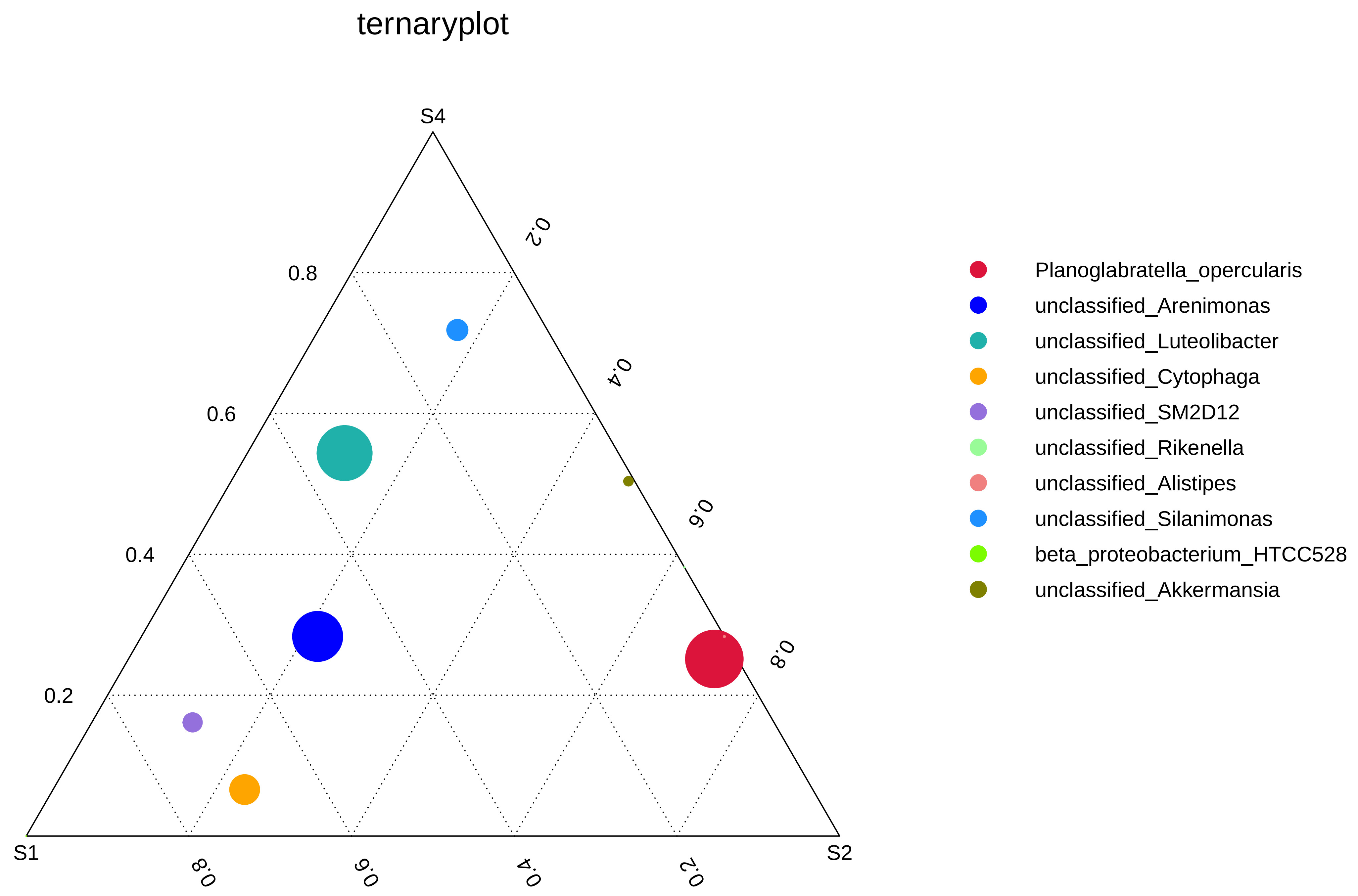
ternaryplot
S4
S1
S2
0.2
0.4
0.6
0.8
0.2
0.4
0.6
0.8
0.8
0.6
0.4
0.2
Planoglabratella_opercularis
unclassified_Arenimonas
unclassified_Luteolibacter
unclassified_Cytophaga
unclassified_SM2D12
unclassified_Rikenella
unclassified_Alistipes
unclassified_Silanimonas
beta_proteobacterium_HTCC528
unclassified_Akkermansia
